# Frailty Status and All-Cause Mortality in the Community-Dwelling Older People: An Umbrella Review

**DOI:** 10.1093/geroni/igaa057.1580

**Published:** 2020-12-16

**Authors:** A R M Saifuddin Ekram, Joanne Ryan, Carlene Britt, Sara Espinoza, Robyn Woods

**Affiliations:** 1 ASPREE, Monash University, Prahran, Victoria, Australia; 2 School of Public Health and Preventive Medicine, Prahran, Victoria, Australia; 3 Monash School of Public Health and Preventive Medicine, Prahran, Victoria, Australia; 4 University of Texas Health Science Center at San Antonio, San Antonio, Texas, United States

## Abstract

Frailty is increasingly recognised for its association with adverse health outcomes including mortality. However, various measures are used to assess frailty, and the strength of association could vary depending on the specific definition used. This umbrella review aimed to map which frailty scale could best predict the relationship between frailty and all-cause mortality among community-dwelling older people. According to the PRISMA guidelines, Medline, Embase, EBSCOhost and Web of Science databases were searched to identify eligible systematic reviews and meta-analyses which examined the association between frailty and all-cause mortality in the community-dwelling older people. Relevant data were extracted and summarised qualitatively. Methodological quality was assessed by AMSTAR-2 checklist. Five moderate-quality systematic reviews with a total of 374,529 participants were identified. Of these, two examined the frailty phenotype and its derivatives, two examined the cumulative deficit models and the other predominantly included studies assessing frailty with the FRAIL scale. All of the reviews found a significant association between frailty status and all-cause mortality. The magnitude of association varied between individual studies, with no consistent pattern related to the frailty measures that were used. In conclusion, regardless of the measure used to assess frailty status, it is associated with an increased risk of all-cause mortality.

